# Obesity-Associated Abdominal Elephantiasis

**DOI:** 10.1155/2013/626739

**Published:** 2013-03-27

**Authors:** Ritesh Kohli, Vivian Argento, Yaw Amoateng-Adjepong

**Affiliations:** ^1^Department of Internal Medicine, Bridgeport Hospital, Yale University School of Medicine, Columbia Tower, Appt. no. 308, 50 Ridgefield Avenue, Bridgeport, CT 06610, USA; ^2^Geriatric Fellowship, Bridgeport Hospital, Yale University School of Medicine, 267 Grant Street, CT 06610, USA; ^3^Department of Internal Medicine, Bridgeport Hospital, Yale University School of Medicine, 267 Grant Street, CT 06610, USA

## Abstract

Abdominal elephantiasis is a rare entity. Abdominal elephantiasis is an uncommon, but deformative and progressive cutaneous disease caused by chronic lymphedema and recurrent streptococcal or *Staphylococcus* infections of the abdominal wall. We present 3 cases of patients with morbid obesity who presented to our hospital with abdominal wall swelling, thickening, erythema, and pain. The abdominal wall and legs were edematous, with cobblestone-like, thickened, hyperpigmented, and fissured plaques on the abdomen. Two patients had localised areas of skin erythema, tenderness, and increased warmth. There was purulent drainage from the abdominal wall in one patient. They were managed with antibiotics with some initial improvement. Meticulous skin care and local keratolytic treatment for the lesions were initiated with limited success due to their late presentation. All three patients refused surgical therapy. *Conclusion*. Early diagnosis is important for the treatment of abdominal elephantiasis and prevention of complications.

## 1. Introduction

Lymphedema refers to excessive lymphatic accumulation in the interstitial space and occurs as a result of inadequate drainage. This may result from intraluminal or extraluminal obstruction, and rarely from congenital hypoplasia of the lymph vessels. When the lymphedema persists, there is often fibrous tissue proliferation and the affected area becomes hard and no longer pits on pressure. Elephantiasis is localised lymphedema with superadded bacterial infection leading to chronic skin changes. It is characterized by chronic edematous and thickened skin resulting from the repeated inflammation of the skin and subcutaneous tissue. We present three cases of abdominal elephantiasis in morbidly obese patients.

**Case 1**

**Figure 1:**
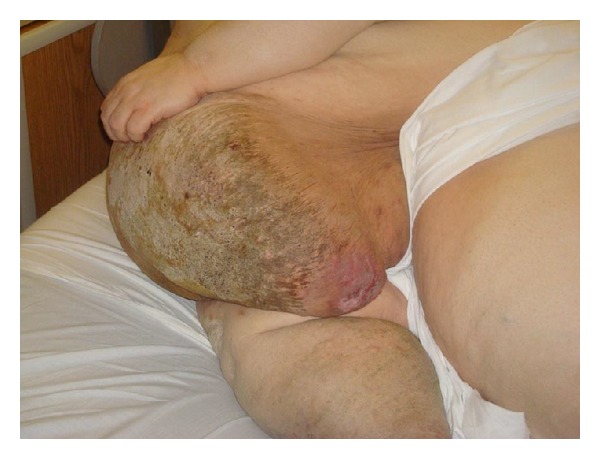
Case 1.

A 53-year-old, morbidly obese gentleman, with a weight of 725 pounds and BMI of 115, who had been homebound for the past 4 years, presented with worsening abdominal swelling and abdominal wall pain. He noticed progressive increases in the thickness of the abdominal skin over the preceding 2 years. His past medical history was significant for type 2 diabetes mellitus, hypothyroidism, recurrent leg cellulitis, and obstructive sleep apnoea. He had no history of congestive heart failure, cancer, liver, or renal disease. He has had no prior surgery or radiation therapy. His physical exams were significant for diffuse, edematous abdominal wall, and legs. The abdominal skin was thickened, fissured, erythematous, and cobblestone-like in appearance (Figure 1). It was warm and tender. There was no visible drainage, nor areas of fluctuance. He had elevated white blood cell count of 13,000. Swab from the fissured areas of the skin grew coagulase negative *Staphylococcus*, *Corynebacterium,* and *Streptococcus* viridians. The polymicrobial growth was likely from colonization/contamination from normal skin flora.

**Case 2**

**Figure 2:**
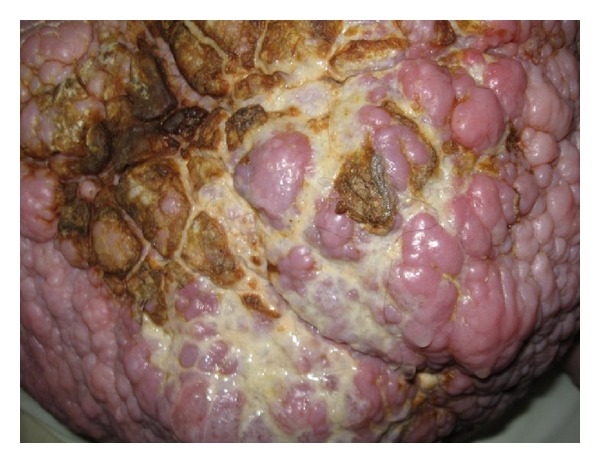
Case 2.

A 60-year-old woman with past medical history of multiple sclerosis, depression, and morbid obesity (with a BMI of 94), presented with abdominal pain and purulent discharge from the abdominal wall. The abdomen was markedly distended and sagging. The abdominal wall was edematous, thickened, hyperpigmented, and excoriated at many sites (Figure 2). There was purulent drainage from the excoriated sites.

**Case 3**

A 80-year-old Caucasian female with morbid obesity (BMI of 55) was admitted with worsening shortness of breath and progressive abdominal and leg swelling. Her past medical history was significant for congestive heart failure, hypertension, chronic kidney disease, and morbid obesity. Her outpatient medications included Lasix, aspirin, and statin. Clinical examination was remarkable for thickened skin of the abdominal wall, fissuring, and nonpitting edema of the abdominal wall. Her legs were visiblly swollen and puffy but were nonpitting. Her lungs were clear of auscultation. Her cardiac echo revealed markedly enlarged right atrium and right ventricle systolic pressure with pulmonary artery hypertension (RVSP = 59 mm Hg, TR Max PG = 49 mm Hg). She was started on dobutamine drip, intravenous Nesiritide, and Lasix. Despite the aggressive diuresis, there was only very minimal decrease in the leg swelling.

## 2. Discussion

 Abdominal elephantiasis is relatively uncommon in the general population. It is relatively more common in the morbidly obese patients [1].

Lymphedema may be classified as primary or secondary. Primary lymphedema results from congenital abnormality or dysfunction of the lymphatic vessels. Secondary lymphedema develops as a consequence of destruction or obstruction of the lymphatic channels. This may result from obesity, infection, trauma, surgery, tumor obstruction, radiation therapy, or Kaposi sarcoma [2, 3].

Morbid obesity can be associated with massive localized lymphedema [1]. It causes excessive adipose tissue deposition that can impair lymphatic drainage and lead to the build-up of protein-rich lymphedema and associated fibrosis and inflammation [4]. In the early stages of lymphedema, impaired lymphatic drainage results in protein rich fluid accumulation in the interstitial compartment manifesting clinically as soft pitting oedema. Later on accumulation of fibroblasts, adipocytes and macrophages in the affected tissue lead to skin thickening and nonpitting oedema. In chronic and advanced stages, local inflammatory response and recurrent infections result in excessive subcutaneous fibrosis and scarring. It is hypothesized that the massiveness of the panniculus causes increased interstitial and intravascular pressure predisposing the patients to chronic low-grade cellulitis and lymphangitis [5–7].

Elephantiasis is an uncommon, but deformative and progressive cutaneous disease caused by chronic lymphedema and recurrent streptococcal or *Staphylooccus* infections [5]. The microorganisms may gain entry into the lymphatics through minor injuries, interdigital fissures, or tinea pedis. Recurrence is common due to the protein rich edema. In chronic lymphedema, the skin becomes markedly thickened, oedematous resulting in severely deformed fibrotic enlargement of the abdomen. There is marked hyperkeratosis with visible papillomatous changes. The epidermis of the abdomen becomes cobblestone with crusting which frequently becomes colonized with multiple bacteria and fungi. Deep folds and cervices are susceptible to formation of fissures and development of recurrent cellulites which may present with localised erythematous changes, warm skin, and pustular exudates.

Abdominal elephantiasis is a rare entity. There are only few reported cases of this abnormality in the medical literature. Thyroid dermopathy, venous stasis dermatitis, and filariasis must be considered in the differential diagnosis. The possibility of filariasis is unlikely in the United States (US) [8]. Filarial disease as a cause of lymphedema is common in sub-Saharan Africa, India, Southeast Asia, parts of South America, the Caribbean, and the South Pacific. Serology studies or the presence of microfilaria in the blood can be used to confirm the diagnosis of filariasis [8]. Venous stasis dermatitis of the abdomen is usually accompanied by swelling of lower limbs. It presents with pitting oedema, associated with erythematous, scaly, pruritic patches, and hemosiderin-stained skin discoloration [9]. Thyroid dermopathy of the abdomen is characterised by lesions that are classically raised, waxy, flesh coloured or yellowish brown, at times accompanied by hyperpigmentation, hyperkeratosis, and hyperhidrosis. In extreme cases, they are characterised by massive nonpitting oedema, accompanied by skin fibrosis and verrucous nodule formation [10]. These cases are difficult to differentiate from chronic lymphedema, and skin biopsy is recommended in these selected cases. Early lymphoedema usually pits but chronic lymphoedema is characterised by nonpitting oedema with skin changes characterised by warty texture (hyperkeratosis) with papillomatosis and induration. 

Distinguishing pure abdominal wall cellulitis from abdominal wall elephantiasis in obese patients can be challenging. Often the two coexist as abdominal wall elephantiasis predisposes the patient to recurrent cellulitis. A long standing, slowo-l progression course and a history of unsuccessful treatment with antibiotics may suggest underlying lymphedema. The diagnosis can be difficult to confirm. Surgery (exeresis/plasty) may confirm the presence of dilated lymph vessels. 

The management of this chronic and deformative disease includes antibiotics to treat cellulitis, topical keratolytics, mechanical massages, oral retinoid, topical benzopyrones [11], and surgical measures. Skin care plays a very important role in management, and patients are instructed to inspect the affected skin on the abdomen daily, with special attention paid to skinfolds, where maceration can occur. If there is marked hyperkeratosis, agents such as 5% salicylic acid should be used, and if skin is dry and flaky, then liquid paraffin should be added. Diuretics are of no value in chronic lymphoedema and their chronic use is associated with side effects and should be avoided. Surgical treatment involves partial lipectomy, debridement of the lesions, and lymphovenous or lymphatic anastomosis [12, 13]. However, wound healing complications are prevalent in these cases. Abdominal lipectomy has proven successful in some cases [13, 14]. We hypothesise that lipectomy in early stages would definitely improve outcome and prevent complications, although limited data is available presently and should be a subject of subsequent development in the near future.

All the 3 patients refused surgical intervention and preferred conservative management; so they were started on antibiotic treatment in Cases 1 and 2, with limited success and so antibiotic was later discontinued. Topical keratolytics were applied in these cases, with transient success as these cases presented so late in the course of disease. In all of our cases, morbid obesity and subsequent chronic lymphedema were considered as the major predisposing factor for their abdominal elephantiasis. 

## 3. Conclusion 

We have presented three cases of presumed abdominal elephantiasis and reviewed the current literature on the subject. Massive, chronic lymphoedema in obese patients can cause extreme disfigurement and physical disability affecting them on both social and physical levels. Management of the morbidly obese patient with lymphedema requires that the obesity be addressed in a frank and supportive way. Treatment of lymphedema must be linked to the treatment of obesity if long-term success is to be achieved. Physicians should be aware of the great risk and complications of chronic lymphoedema, strive to develop a collaborative approach to care, and be prepared to educate and closely monitor these patients. Management in advanced stages is usually unsuccessful. The initial stages of this condition can be treated more easily with topical therapies. Early diagnosis is important for the treatment and prevention of complication.
